# Crystal structures of two hydrazide derivatives of mefenamic acid, 3-(2,3-di­methyl­anilino)-*N*′-[(*E*)-(furan-2-yl)methyl­idene]benzohydrazide and *N*′-[(*E*)-benzyl­idene]-2-(2,3-di­methyl­anilino)benzo­hydrazide

**DOI:** 10.1107/S2056989021001353

**Published:** 2021-02-12

**Authors:** Shaaban K. Mohamed, Joel T. Mague, Mehmet Akkurt, Mustafa R. Albayati, Sahar M. I. Elgarhy, Elham A. Al-Taifi

**Affiliations:** a Chemistry and Environmental Division, Manchester Metropolitan University, Manchester, M1 5GD, England; b Chemistry Department, Faculty of Science, Minia University, 61519 El-Minia, Egypt; c Department of Chemistry, Tulane University, New Orleans, LA 70118, USA; d Department of Physics, Faculty of Sciences, Erciyes University, 38039 Kayseri, Turkey; e Chemistry Department, College of Education, Kirkuk University, Kirkuk, Iraq; f Faculty of Science, Department of Bio-Chemistry, Beni Suef University, Beni Suef, Egypt; g Department of Chemistry, Faculty of Science, Sana’a University, Sana’a, Yemen

**Keywords:** crystal structure, hydrogen bond, benzohydrazide, C—H⋯π(ring), mefenamic, NSAIDs

## Abstract

The conformation about the central benzene ring in the mol­ecule of (I), C_20_H_19_N_3_O_2_, is partially determined by an intra­molecular N—H⋯O hydrogen bond. In the crystal, chains parallel to the *c* axis are generated by inter­molecular N—H⋯O hydrogen bonds with the chains assembled into a three-dimensional network structure by inter­molecular C—H⋯O hydrogen bonds and C—H⋯π(ring) inter­actions. The mol­ecule of (II), C_22_H_21_N_3_O, differs from (I) only in the substituent at the hydrazide N atom where a phenyl­methyl­ene moiety for (II) is present instead of a furan­methyl­ene moiety for (I). Hence, mol­ecules of (I) and (II) show similarities in their mol­ecular and crystal structures. The conformation of the central portion of the mol­ecule of (II) is also therefore partially determined by an intra­molecular N—H⋯O hydrogen bond and inter­molecular N—H⋯O hydrogen bonds form chains parallel to the *c* axis. Likewise, the chains are connected into a three-dimensional network by C—H⋯O hydrogen bonds and C—H⋯π(ring) inter­actions.

## Abstract 2

The mol­ecular and crystal structures of (I), C_20_H_19_N_3_O_2_, and (II), C_22_H_21_N_3_O, are similar because they differ only in the substituent at the hydrazide N atom where a phenyl­methyl­ene moiety for (II) is present instead of a furan­methyl­ene moiety for (I).

## Chemical context   

Hydrazones possess a wide variety of biological activities such as anti­convulsant (Kumar *et al.*, 2010 ▸), anti-depressant (Mohareb *et al.*, 2010 ▸), analgesic, anti-inflammatory (Hernandez *et al.*, 2012 ▸), anti­microbial (Maguene *et al.*, 2011 ▸), anti­cancer (Al-Said *et al.*, 2011 ▸) or anti­parasitic (Siddiqui *et al.*, 2012 ▸) properties. A better tolerated and potent non-steroidal anti-inflammatory drug (NSAID) with fewer side effect characteristic is mefenamic acid. This drug belongs to the most commonly prescribed medications worldwide for treatment of painful inflammatory conditions such as rheumatic arthritis, traumatic injuries, pain and fever (Abbas, 2017 ▸). It is also used to treat mild to moderate pain, including menstrual pain and the associated migraines (Pringsheim *et al.*, 2008 ▸). With this background in mind, we report here the synthesis and crystal structural determination of two hydrazide derivatives of mefenamic acid, (I) and (II).
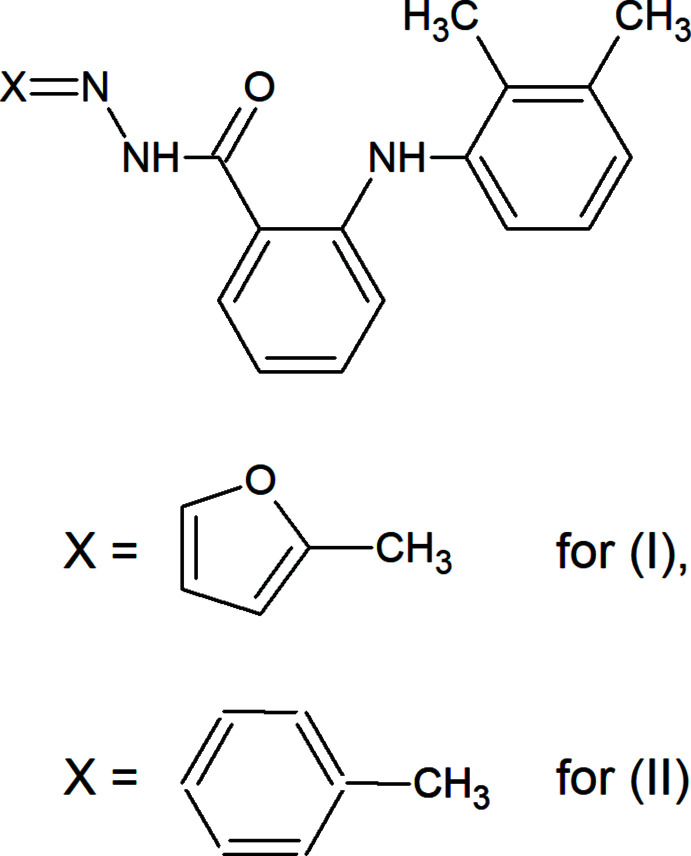


## Structural commentary   

**Figure 1:**
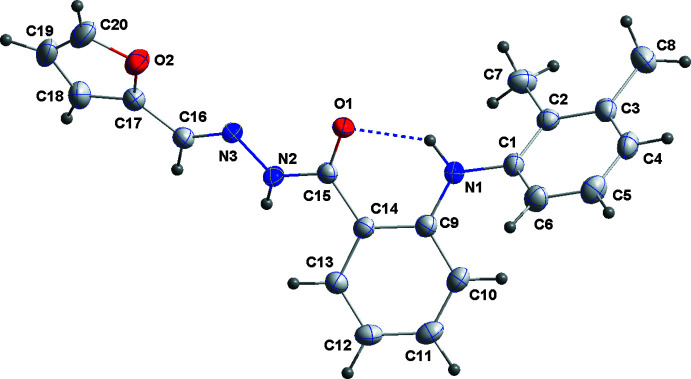
The mol­ecule of (I) with atom-labeling scheme and displacement ellipsoids drawn at the 50% probability level. The intra­molecular N—H⋯O hydrogen bond is shown as a dashed line.

In the mol­ecule of (I) (Fig. 1 ▸), the dihedral angles between the central C9–C14 benzene ring and the C1–C6 and C17–C20/O2 rings are, respectively, 51.90 (6) and 43.32 (8)°. The conformation about the central portion of the mol­ecule is partially determined by the intra­molecular N1—H1⋯O1 hydrogen bond (Table 1 ▸).

**Figure 2:**
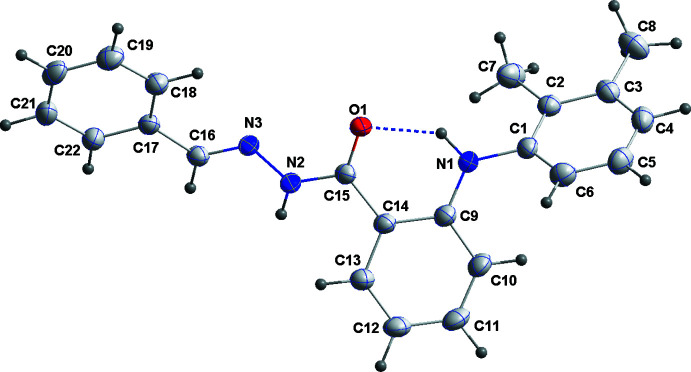
The mol­ecule of (II) with atom-labeling scheme and displacement ellipsoids drawn at the 50% probability level. The intra­molecular N—H⋯O hydrogen bond is shown by a dashed line.

Mol­ecule (II) (Fig. 2 ▸) differs from mol­ecule (I) only by the substituent at N3, *i.e.* a phenyl­methyl­ene moiety for (II) instead of a furan­methyl­ene moiety for (I). Hence, the structural characteristics for most parts of the two mol­ecules are very similar, as exemplified by the dihedral angles between the central C9–C14 benzene ring and the C1–C6 and C17–C22 benzene rings of 57.38 (6) and 43.48 (6)°, respectively, observed in mol­ecule (II). Likewise, in the crystal of (II), the conformation of the central portion of the mol­ecule is also partially determined by the intra­molecular N1—H1⋯O1 hydrogen bond (Table 2 ▸; Fig. 2 ▸).

## Supra­molecular features   

**Figure 3:**
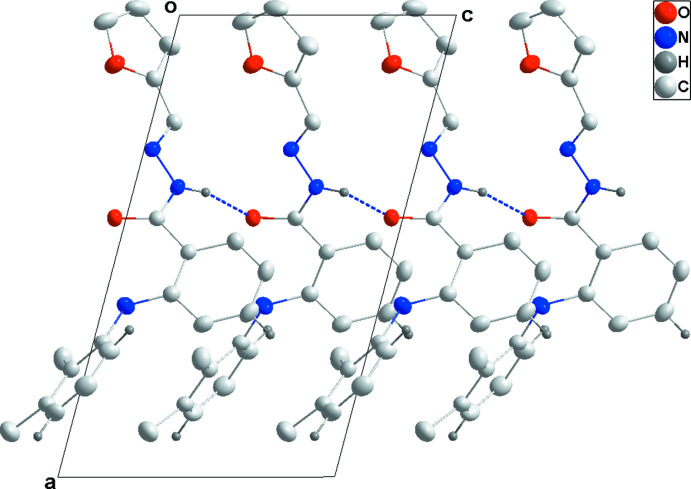
A portion of one N—H⋯O hydrogen-bonded chain viewed along the *b* axis of (I) with hydrogen bonds shown as dashed lines.

**Figure 4:**
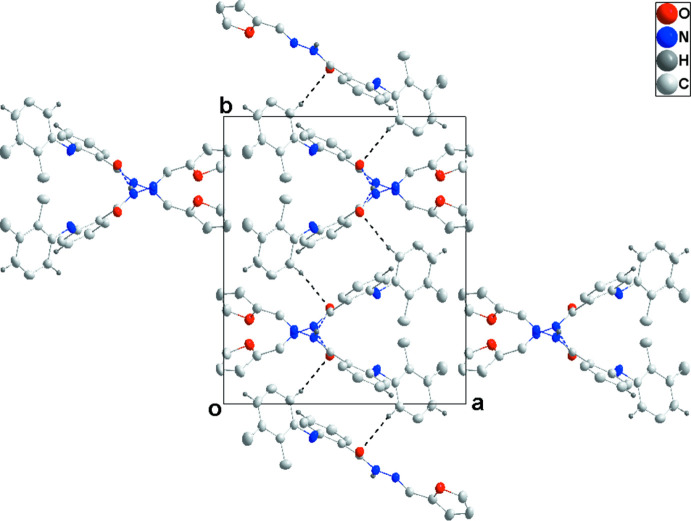
Packing view of (I) along the *c* axis with inter­molecular C—H⋯O hydrogen bonds shown as dashed lines.

In the crystal structure of (I), chains of mol­ecules extending parallel to the *c-*axis direction are generated by N2—H2⋯O2 hydrogen bonds (Table 1 ▸; Fig. 3 ▸). These chains are linked into a three-dimensional network structure by a combination of C6—H6⋯O1 hydrogen bonds and C4—H4⋯*Cg*1 and C11—H11⋯*Cg*2 inter­actions (Table 1 ▸; Fig. 4 ▸).

**Figure 5:**
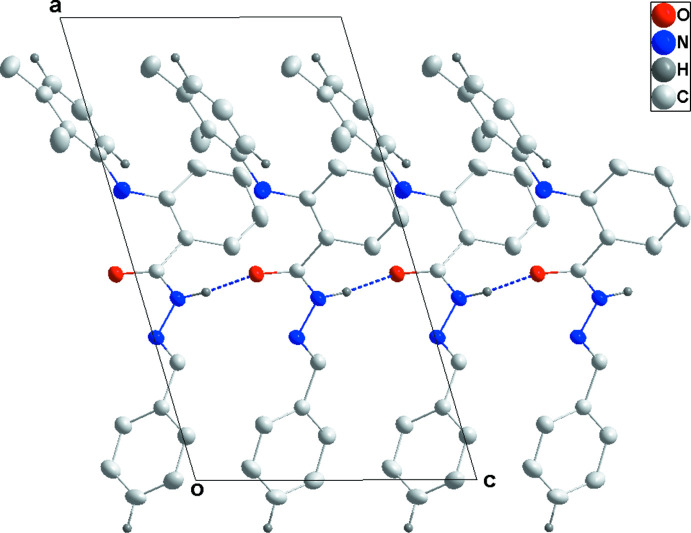
A portion of the N—H⋯O hydrogen-bonded chain viewed along the *b* axis of (II) with hydrogen bonds shown as dashed lines..

**Figure 6:**
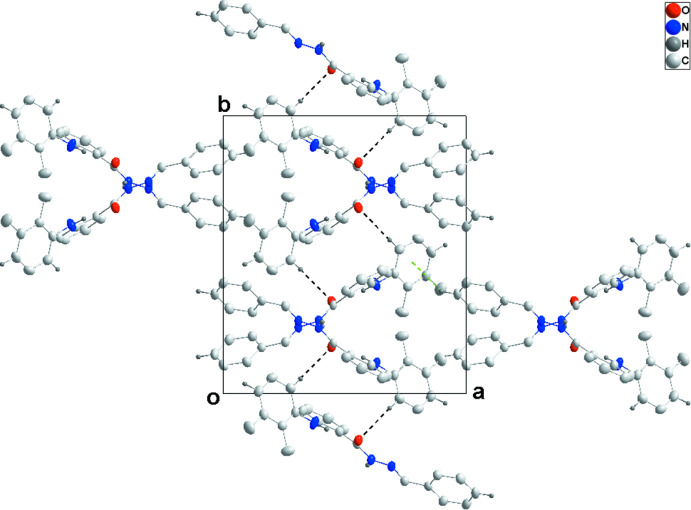
Packing view of (II) along the *c* axis with inter­molecular C—H⋯O hydrogen bonds shown as dashed lines.

In the crystal structure of (II), inter­molecular N2—H2⋯O1 hydrogen bonds form chains parallel the *c-*axis direction (Table 2 ▸; Fig. 5 ▸), which are connected through C6—H6⋯O1 hydrogen bonds and C4—H4⋯*Cg*3 and C20—H20⋯*Cg*1 inter­actions to form a three-dimensional network (Table 2 ▸; Fig. 6 ▸).

## Database survey   

A search of the Cambridge Structural Database (CSD, version 5.42, November 2020; Groom *et al.*, 2016 ▸) gave six hits for structures with a 2-(2,3-di­methyl­anilino)-*N*′-methyl­idene­benzohydrazide skeleton: *N*′-[(4-chloro­phen­yl)methyl­idene]-2-[(2,3-di­methyl­phen­yl)amino]­benzohydrazide (VEDBAK; Jasinski *et al.*, 2017 ▸), *N*′-[1-(4-chloro­phen­yl)ethyl­idene]-2-[(2,3-di­methyl­phen­yl)amino]­benzohydrazide (LEBSET; Mohamed *et al.*, 2017 ▸), 2-[(2,3-di­methyl­phen­yl)amino]-*N*′-(2-hy­droxy­benzyl­idene)benzohydrazide (DABREG; Mohamed *et al.*, 2015 ▸), 2-[(2,3-di­methyl­phen­yl)amino]-*N*′-(2-thienyl­methyl­ene)benzohydrazide (LEGHAI; Fun *et al.*, 2012*a* ▸), 2-[(2,3-di­methyl­phen­yl)amino]­benzohydrazide (LEGHIQ; Fun *et al.*, 2012*b* ▸) and (*E*)-2-[(2,3-di­methyl­phen­yl)amino]-*N*′-(2-methyl-5-(prop-1-en-2-yl)cyclo­hex-2-en-1-yl­idene)benzo­hydra­zide (YAXJUE; Bhat *et al.*, 2012 ▸).

In the structure of VEDBAK, the dihedral angle between the planes of the chloro­phenyl and di­methyl­phenyl rings is 66.50 (9)°. These rings make dihedral angles of 47.79 (8) and 69.24 (9)°, respectively, with the central benzene ring. In the crystal structure of VEDBAK, mol­ecules are linked into a three-dimensional supra­molecular network by N—H⋯O, C—H⋯O hydrogen bonds and weak C—H⋯π inter­actions.

In the crystal structure of LEBSET, mol­ecules are linked into a three-dimensional supra­molecular network by N—H⋯N, N—H⋯O, C—H⋯O hydrogen bonds and weak C—H⋯π inter­actions.

The asymmetric unit of DABREG consists of two mol­ecules (*A* and *B*) having differing conformations that mainly concern the dihedral angles between the hy­droxy­phenyl and di­methyl­phenyl rings relative to the central phenyl­ene ring, with values of 30.16 (6) and 58.60 (6)° in mol­ecule *A* and of 13.42 (7) and 60.31 (7)° in mol­ecule *B*. With the exception of the di­methyl­phenyl substituent, the conformations of the rest of each mol­ecule are largely determined by intra­molecular O—H⋯N and N—H⋯O hydrogen bonds. In the crystal structure, N—H⋯O hydrogen bonds link the mol­ecules into chains extending parallel to the *a* axis where the types of mol­ecules alternate in an ⋯*A*⋯*B*⋯*A*⋯*B*⋯ fashion.

In LEGHAI, the central benzene ring makes dihedral angles of 45.36 (9) and 55.33 (9)° with the thio­phene ring and the dimethyl-substituted benzene ring, respectively. The dihedral angle between the thio­phene ring and dimethyl-substituted benzene ring is 83.60 (9)°. The thio­phene ring and the benzene ring are twisted from the mean plane of the C(=O)—N—N=C bridge [maximum deviation = 0.0860 (13) Å], with dihedral angles of 23.86 (9) and 24.77 (8)°, respectively. An intra­molecular N—H⋯O hydrogen bond generates an *S*(6) ring motif. In the crystal structure of LEGHAI, mol­ecules are linked by N—H⋯O and C—H⋯O hydrogen bonds to the same acceptor atom, forming sheets lying parallel to the *bc* plane. The crystal packing also features C—H⋯π inter­actions.

In LEGHIQ, the dihedral angle between the benzene rings is 58.05 (9)°. The non-H atoms of the hydrazide group lie in a common plane (r.m.s. deviation = 0.0006 Å) and are close to co-planar with their attached benzene ring [dihedral angle = 8.02 (9)°]. An intra­molecular N—H⋯O hydrogen bond generates an *S*(6) ring motif in the mol­ecule, and a short intra­molecular contact (H⋯H = 1.88 Å) is also observed. In the crystal structure of LEGHIQ, mol­ecules are linked by pairs of N—H⋯N hydrogen bonds into inversion dimers. The crystal packing also features C—H⋯π inter­actions.

The asymmetric unit of the compound YAXJUE comprises two mol­ecules. The dihedral angles between the benzene rings in the two mol­ecules are 59.7 (2) and 61.27 (18)°. The cyclo­hexene rings adopt sofa and half-chair conformations. In the crystal structure of YAXJUE, mol­ecules are connected *via* N—H⋯O and weak C—H⋯O hydrogen bonds, forming chains along the *a*-axis direction. In each mol­ecule, there is an intra­molecular N—H⋯O hydrogen bond.

## Synthesis and crystallization   

**Synthesis of (I):** A mixture of 1 mmol of 2-furaldehyde (96 mg) and 1 mmol of 2-[(2,3-di­methyl­phen­yl)amino]­benzohydrazide (255 mg) in 20 ml of ethanol was refluxed and monitored by TLC until completion. The reaction mixture was cooled to room temperature when the solid product was obtained. The crude product was filtered off, dried and recrystallized from ethanol to afford crystals suitable for X-ray diffraction. M.p. 479–483 K.

**Synthesis of (II):** In a solution of 20 ml of ethanol, a mixture of 106 mg (1 mmol) of benzaldehyde (106 mg) and 255 mg (1 mmol) of 2-[(2,3-di­methyl­phen­yl)amino]­benzohydrazide was refluxed for 4 h. The solid product was obtained after the reaction mixture was cooled to room temperature. The crude product was filtered off, dried and recrystallized from ethanol to afford crystals suitable for X-ray diffraction. M.p. 466–469 K.

## Refinement   

Crystal data, data collection and structure refinement details are summarized in Table 3 ▸. For (I) and (II), all H atoms were located in a difference-Fourier map and were refined freely.

## Supplementary Material

Crystal structure: contains datablock(s) global, I, II. DOI: 10.1107/S2056989021001353/wm5599sup1.cif

Structure factors: contains datablock(s) I. DOI: 10.1107/S2056989021001353/wm5599Isup2.hkl

Structure factors: contains datablock(s) II. DOI: 10.1107/S2056989021001353/wm5599IIsup3.hkl

Supporting information file. DOI: 10.1107/S2056989021001353/wm5599Isup4.cml

Supporting information file. DOI: 10.1107/S2056989021001353/wm5599IIsup5.cml

CCDC references: 2061393, 2061392

Additional supporting information:  crystallographic information; 3D view; checkCIF report

## Figures and Tables

**Table 1:** Hydrogen-bond geometry (Å, °) for (I) *Cg*1 and *Cg*2 are the centroids of the C17–C20/O2 and C1–C6 rings, respectively.

*D*—H⋯*A*	*D*—H	H⋯*A*	*D*⋯*A*	*D*—H⋯*A*
N1—H1⋯O1	0.910 (19)	1.977 (18)	2.7045 (14)	135.8 (15)
N2—H2⋯O1^i^	0.883 (18)	2.014 (18)	2.8458 (13)	156.4 (15)
C4—H4⋯*Cg*1^ii^	0.955 (17)	2.941 (17)	3.7248 (15)	140.1 (17)
C6—H6⋯O1^iii^	0.976 (18)	2.556 (18)	3.3434 (16)	137.7 (14)
C11—H11⋯*Cg*2^iv^	0.996 (16)	2.765 (16)	3.6231 (14)	144.8 (12)

Symmetry codes: (i) x, -y+{\script{1\over 2}}, z+{\script{1\over 2}}; (ii) -x+1, y+{\script{1\over 2}}, -z+{\script{1\over 2}}; (iii) -x+1, -y+1, -z+1; (iv) x, y, z+1.

**Table 2:** Hydrogen-bond geometry (Å, °) for (II) *Cg*1 and *Cg*3 are the centroids of the C1–C6 and C17–C22 benzene rings, respectively.

*D*—H⋯*A*	*D*—H	H⋯*A*	*D*⋯*A*	*D*—H⋯*A*
N1—H1⋯O1	0.908 (18)	1.946 (18)	2.6920 (13)	138.2 (15)
N2—H2⋯O1^i^	0.913 (16)	1.974 (17)	2.8564 (13)	162.0 (14)
C4—H4⋯*Cg*3^ii^	1.001 (17)	2.796 (17)	3.6141 (15)	139.4 (13)
C6—H6⋯O1^iii^	0.980 (18)	2.583 (19)	3.4815 (16)	152.5 (13)
C20—H20⋯*Cg*1^iv^	1.000 (18)	2.838 (17)	3.6644 (15)	140.5 (13)

Symmetry codes: (i) x, -y+{\script{1\over 2}}, z+{\script{1\over 2}}; (ii) -x+1, y+{\script{1\over 2}}, -z+{\script{1\over 2}}; (iii) -x+1, -y+1, -z+1; (iv) x-1, -y-{\script{1\over 2}}, z-{\script{3\over 2}}.

**Table 3:** Experimental details

	(I)	(II)
Crystal data		
Chemical formula	C_20_H_19_N_3_O_2_	C_22_H_21_N_3_O
*M*_r_	333.38	343.42
Crystal system, space group	Monoclinic, *P*2_1_/*c*	Monoclinic, *P*2_1_/*c*
Temperature (K)	150	150
*a*, *b*, *c* (Å)	13.8467 (3), 15.8409 (3), 8.0225 (2)	14.3493 (8), 15.7501 (9), 8.3737 (5)
β (°)	104.814 (1)	106.285 (2)
*V* (Å^3^)	1701.20 (7)	1816.55 (18)
*Z*	4	4
Radiation type	Cu *K*α	Cu *K*α
μ (mm^−1^)	0.69	0.62
Crystal size (mm)	0.19 × 0.11 × 0.07	0.19 × 0.13 × 0.08

Data collection		
Diffractometer	Bruker D8 VENTURE PHOTON 100 CMOS	Bruker D8 VENTURE PHOTON 100 CMOS
Absorption correction	Multi-scan (*SADABS*; Krause *et al.*, 2015 ▸)	Multi-scan (*SADABS*; Krause *et al.*, 2015 ▸)
*T*_min_, *T*_max_	0.88, 0.95	0.86, 0.95
No. of measured, independent and observed [*I* > 2σ(*I*)] reflections	13222, 3372, 2939	13875, 3665, 3140
*R*_int_	0.031	0.031
(sin θ/λ)_max_ (Å^−1^)	0.625	0.625

Refinement		
*R*[*F* ^2^ > 2σ(*F* ^2^)], *wR*(*F* ^2^), *S*	0.036, 0.095, 1.05	0.037, 0.092, 1.04
No. of reflections	3372	3665
No. of parameters	303	320
H-atom treatment	All H-atom parameters refined	All H-atom parameters refined
Δρ_max_, Δρ_min_ (e Å^−3^)	0.23, −0.19	0.17, −0.16

Computer programs: *APEX3* and *SAINT* (Bruker, 2016 ▸), *SHELXT* (Sheldrick, 2015*a* ▸), *SHELXL2016/6* (Sheldrick, 2015*b* ▸), *DIAMOND* (Brandenburg & Putz, 2012 ▸) and *publCIF* (Westrip, 2010 ▸).

## supplementary crystallographic information

### 3-(2,3-Dimethylanilino)-N'-[(E)-(furan-2-yl)methylidene]benzohydrazide (I) Crystal data

**Table:**

C_20_H_19_N_3_O_2_	*F*(000) = 704
*M**_r_* = 333.38	*D*_x_ = 1.302 Mg m^−^^3^
Monoclinic, *P*2_1_/*c*	Cu *K*α radiation, λ = 1.54178 Å
*a* = 13.8467 (3) Å	Cell parameters from 9755 reflections
*b* = 15.8409 (3) Å	θ = 3.3–74.5°
*c* = 8.0225 (2) Å	µ = 0.69 mm^−^^1^
β = 104.814 (1)°	*T* = 150 K
*V* = 1701.20 (7) Å^3^	Column, colourless
*Z* = 4	0.19 × 0.11 × 0.07 mm

### 3-(2,3-Dimethylanilino)-N'-[(E)-(furan-2-yl)methylidene]benzohydrazide (I) Data collection

**Table:**

Bruker D8 VENTURE PHOTON 100 CMOS diffractometer	3372 independent reflections
Radiation source: INCOATEC IµS micro–focus source	2939 reflections with *I* > 2σ(*I*)
Mirror monochromator	*R*_int_ = 0.031
Detector resolution: 10.4167 pixels mm^-1^	θ_max_ = 74.5°, θ_min_ = 3.3°
ω scans	*h* = −14→16
Absorption correction: multi-scan (*SADABS*; Krause *et al.*, 2015)	*k* = −18→19
*T*_min_ = 0.88, *T*_max_ = 0.95	*l* = −9→9
13222 measured reflections	

### 3-(2,3-Dimethylanilino)-N'-[(E)-(furan-2-yl)methylidene]benzohydrazide (I) Refinement

**Table:**

Refinement on *F*^2^	Secondary atom site location: difference Fourier map
Least-squares matrix: full	Hydrogen site location: difference Fourier map
*R*[*F*^2^ > 2σ(*F*^2^)] = 0.036	All H-atom parameters refined
*wR*(*F*^2^) = 0.095	*w* = 1/[σ^2^(*F*_o_^2^) + (0.0475*P*)^2^ + 0.3981*P*] where *P* = (*F*_o_^2^ + 2*F*_c_^2^)/3
*S* = 1.05	(Δ/σ)_max_ < 0.001
3372 reflections	Δρ_max_ = 0.23 e Å^−^^3^
303 parameters	Δρ_min_ = −0.19 e Å^−^^3^
0 restraints	Extinction correction: *SHELXL* 2016/6 (Sheldrick, 2015*b*), Fc^*^=kFc[1+0.001xFc^2^λ^3^/sin(2θ)]^-1/4^
Primary atom site location: structure-invariant direct methods	Extinction coefficient: 0.0049 (4)

### 3-(2,3-Dimethylanilino)-N'-[(E)-(furan-2-yl)methylidene]benzohydrazide (I) Special details

**Table:**

Geometry. All esds (except the esd in the dihedral angle between two l.s. planes) are estimated using the full covariance matrix. The cell esds are taken into account individually in the estimation of esds in distances, angles and torsion angles; correlations between esds in cell parameters are only used when they are defined by crystal symmetry. An approximate (isotropic) treatment of cell esds is used for estimating esds involving l.s. planes.
Refinement. Refinement of F^2^ against ALL reflections. The weighted R-factor wR and goodness of fit S are based on F^2^, conventional R-factors R are based on F, with F set to zero for negative F^2^. The threshold expression of F^2^ > 2sigma(F^2^) is used only for calculating R-factors(gt) etc. and is not relevant to the choice of reflections for refinement. R-factors based on F^2^ are statistically about twice as large as those based on F, and R- factors based on ALL data will be even larger.

### 3-(2,3-Dimethylanilino)-N'-[(E)-(furan-2-yl)methylidene]benzohydrazide (I) Fractional atomic coordinates and isotropic or equivalent isotropic displacement parameters (Å^2^)

**Table:**

	*x*	*y*	*z*	*U*_iso_*/*U*_eq_	
O1	0.43892 (6)	0.33178 (5)	0.45837 (10)	0.0275 (2)	
O2	0.10644 (7)	0.20592 (6)	0.31256 (12)	0.0371 (2)	
N1	0.62923 (8)	0.38535 (8)	0.58252 (15)	0.0353 (3)	
H1	0.5810 (13)	0.3629 (11)	0.495 (2)	0.051 (5)*	
N2	0.37222 (7)	0.26862 (7)	0.65665 (13)	0.0262 (2)	
H2	0.3836 (12)	0.2485 (11)	0.763 (2)	0.043 (4)*	
N3	0.29029 (7)	0.24278 (6)	0.52997 (12)	0.0260 (2)	
C1	0.70907 (9)	0.42610 (8)	0.53437 (15)	0.0279 (3)	
C2	0.76645 (9)	0.37935 (8)	0.44626 (15)	0.0276 (3)	
C3	0.84199 (9)	0.42133 (9)	0.39033 (15)	0.0313 (3)	
C4	0.85989 (10)	0.50619 (9)	0.42738 (17)	0.0361 (3)	
H4	0.9119 (12)	0.5334 (11)	0.389 (2)	0.047 (5)*	
C5	0.80281 (11)	0.55168 (9)	0.51433 (19)	0.0378 (3)	
H5	0.8130 (13)	0.6122 (12)	0.533 (2)	0.052 (5)*	
C6	0.72631 (10)	0.51191 (9)	0.56546 (18)	0.0342 (3)	
H6	0.6815 (13)	0.5435 (11)	0.618 (2)	0.052 (5)*	
C7	0.74397 (12)	0.28753 (9)	0.40596 (19)	0.0381 (3)	
H7A	0.6963 (16)	0.2816 (13)	0.295 (3)	0.073 (6)*	
H7B	0.8049 (17)	0.2542 (14)	0.398 (3)	0.078 (6)*	
H7C	0.7169 (13)	0.2599 (12)	0.493 (2)	0.055 (5)*	
C8	0.90166 (13)	0.37716 (12)	0.2834 (2)	0.0470 (4)	
H8A	0.8587 (14)	0.3622 (12)	0.170 (3)	0.056 (5)*	
H8B	0.957 (2)	0.4145 (17)	0.265 (3)	0.105 (8)*	
H8C	0.9298 (16)	0.3242 (15)	0.331 (3)	0.075 (6)*	
C9	0.60374 (9)	0.39480 (8)	0.73618 (15)	0.0275 (3)	
C10	0.66889 (10)	0.43330 (8)	0.88024 (16)	0.0326 (3)	
H10	0.7324 (13)	0.4574 (11)	0.866 (2)	0.045 (4)*	
C11	0.64394 (10)	0.44053 (9)	1.03499 (16)	0.0350 (3)	
H11	0.6916 (11)	0.4702 (10)	1.131 (2)	0.037 (4)*	
C12	0.55432 (10)	0.40856 (9)	1.05579 (16)	0.0343 (3)	
H12	0.5341 (11)	0.4161 (10)	1.165 (2)	0.037 (4)*	
C13	0.48990 (10)	0.36959 (8)	0.91722 (16)	0.0293 (3)	
H13	0.4250 (12)	0.3490 (10)	0.9272 (19)	0.038 (4)*	
C14	0.51281 (9)	0.36090 (7)	0.75761 (15)	0.0251 (3)	
C15	0.44003 (8)	0.32000 (7)	0.61193 (14)	0.0238 (2)	
C16	0.23183 (9)	0.19224 (8)	0.58218 (15)	0.0280 (3)	
H16	0.2460 (11)	0.1729 (10)	0.701 (2)	0.038 (4)*	
C17	0.13770 (9)	0.16726 (8)	0.46938 (16)	0.0297 (3)	
C18	0.06614 (11)	0.11334 (10)	0.4926 (2)	0.0410 (3)	
H18	0.0716 (14)	0.0788 (12)	0.597 (2)	0.059 (5)*	
C19	−0.01455 (11)	0.11896 (10)	0.3418 (2)	0.0440 (4)	
H19	−0.0782 (15)	0.0886 (12)	0.319 (2)	0.061 (5)*	
C20	0.01305 (10)	0.17477 (10)	0.2391 (2)	0.0425 (4)	
H20	−0.0217 (15)	0.1968 (13)	0.124 (3)	0.063 (5)*	

### 3-(2,3-Dimethylanilino)-N'-[(E)-(furan-2-yl)methylidene]benzohydrazide (I) Atomic displacement parameters (Å^2^)

**Table:**

	*U*^11^	*U*^22^	*U*^33^	*U*^12^	*U*^13^	*U*^23^
O1	0.0276 (4)	0.0337 (5)	0.0215 (4)	−0.0028 (3)	0.0067 (3)	−0.0006 (3)
O2	0.0308 (5)	0.0430 (5)	0.0328 (5)	−0.0046 (4)	−0.0004 (4)	0.0003 (4)
N1	0.0289 (6)	0.0477 (7)	0.0315 (6)	−0.0131 (5)	0.0118 (4)	−0.0092 (5)
N2	0.0236 (5)	0.0337 (5)	0.0197 (5)	−0.0034 (4)	0.0026 (4)	0.0016 (4)
N3	0.0225 (5)	0.0315 (5)	0.0229 (5)	−0.0018 (4)	0.0040 (4)	−0.0016 (4)
C1	0.0224 (6)	0.0326 (6)	0.0280 (6)	−0.0021 (5)	0.0051 (4)	0.0011 (5)
C2	0.0266 (6)	0.0307 (6)	0.0239 (6)	−0.0007 (5)	0.0037 (4)	0.0010 (5)
C3	0.0236 (6)	0.0426 (7)	0.0269 (6)	−0.0005 (5)	0.0050 (5)	0.0022 (5)
C4	0.0288 (7)	0.0433 (8)	0.0352 (7)	−0.0101 (6)	0.0065 (5)	0.0057 (6)
C5	0.0387 (8)	0.0292 (7)	0.0428 (8)	−0.0044 (5)	0.0056 (6)	0.0025 (6)
C6	0.0295 (7)	0.0321 (7)	0.0402 (7)	0.0025 (5)	0.0078 (5)	−0.0009 (5)
C7	0.0498 (9)	0.0314 (7)	0.0344 (7)	−0.0032 (6)	0.0130 (6)	−0.0027 (5)
C8	0.0392 (9)	0.0645 (11)	0.0414 (9)	−0.0004 (7)	0.0176 (7)	−0.0055 (7)
C9	0.0250 (6)	0.0297 (6)	0.0271 (6)	0.0004 (5)	0.0052 (4)	0.0000 (5)
C10	0.0260 (6)	0.0356 (7)	0.0327 (7)	−0.0025 (5)	0.0011 (5)	−0.0009 (5)
C11	0.0358 (7)	0.0358 (7)	0.0275 (6)	−0.0008 (5)	−0.0028 (5)	−0.0015 (5)
C12	0.0411 (7)	0.0370 (7)	0.0235 (6)	−0.0008 (6)	0.0060 (5)	−0.0024 (5)
C13	0.0301 (7)	0.0313 (6)	0.0268 (6)	−0.0012 (5)	0.0079 (5)	0.0003 (5)
C14	0.0232 (6)	0.0275 (6)	0.0233 (6)	0.0009 (4)	0.0033 (4)	0.0001 (4)
C15	0.0217 (6)	0.0260 (6)	0.0236 (6)	0.0023 (4)	0.0055 (4)	0.0000 (4)
C16	0.0264 (6)	0.0335 (6)	0.0249 (6)	−0.0010 (5)	0.0081 (5)	−0.0001 (5)
C17	0.0272 (6)	0.0341 (6)	0.0286 (6)	−0.0013 (5)	0.0088 (5)	−0.0029 (5)
C18	0.0342 (7)	0.0482 (8)	0.0433 (8)	−0.0102 (6)	0.0150 (6)	−0.0046 (6)
C19	0.0250 (7)	0.0502 (9)	0.0563 (9)	−0.0075 (6)	0.0097 (6)	−0.0170 (7)
C20	0.0285 (7)	0.0471 (8)	0.0451 (8)	−0.0014 (6)	−0.0033 (6)	−0.0108 (7)

### 3-(2,3-Dimethylanilino)-N'-[(E)-(furan-2-yl)methylidene]benzohydrazide (I) Geometric parameters (Å, º)

**Table:**

O1—C15	1.2422 (14)	C7—H7C	0.978 (19)
O2—C17	1.3663 (16)	C8—H8A	0.98 (2)
O2—C20	1.3687 (16)	C8—H8B	1.01 (3)
N1—C9	1.3744 (16)	C8—H8C	0.96 (2)
N1—C1	1.4165 (16)	C9—C10	1.4102 (17)
N1—H1	0.910 (19)	C9—C14	1.4191 (17)
N2—C15	1.3582 (15)	C10—C11	1.3759 (19)
N2—N3	1.3777 (13)	C10—H10	0.993 (17)
N2—H2	0.883 (18)	C11—C12	1.389 (2)
N3—C16	1.2826 (16)	C11—H11	0.996 (16)
C1—C6	1.3915 (18)	C12—C13	1.3804 (18)
C1—C2	1.4023 (17)	C12—H12	0.989 (16)
C2—C3	1.4063 (17)	C13—C14	1.4024 (17)
C2—C7	1.5053 (18)	C13—H13	0.978 (16)
C3—C4	1.385 (2)	C14—C15	1.4829 (15)
C3—C8	1.507 (2)	C16—C17	1.4392 (17)
C4—C5	1.383 (2)	C16—H16	0.973 (16)
C4—H4	0.955 (17)	C17—C18	1.3566 (19)
C5—C6	1.3818 (19)	C18—C19	1.424 (2)
C5—H5	0.975 (19)	C18—H18	0.990 (19)
C6—H6	0.976 (18)	C19—C20	1.330 (2)
C7—H7A	0.97 (2)	C19—H19	0.98 (2)
C7—H7B	1.01 (2)	C20—H20	0.99 (2)
			
C17—O2—C20	106.08 (11)	H8B—C8—H8C	108.9 (19)
C9—N1—C1	126.32 (11)	N1—C9—C10	121.67 (11)
C9—N1—H1	115.6 (11)	N1—C9—C14	120.45 (11)
C1—N1—H1	115.8 (11)	C10—C9—C14	117.77 (11)
C15—N2—N3	118.53 (10)	C11—C10—C9	121.32 (12)
C15—N2—H2	120.4 (11)	C11—C10—H10	120.6 (10)
N3—N2—H2	120.9 (11)	C9—C10—H10	118.0 (10)
C16—N3—N2	114.45 (10)	C10—C11—C12	121.07 (12)
C6—C1—C2	120.85 (11)	C10—C11—H11	118.0 (9)
C6—C1—N1	120.43 (11)	C12—C11—H11	120.9 (9)
C2—C1—N1	118.62 (11)	C13—C12—C11	118.64 (12)
C1—C2—C3	118.33 (11)	C13—C12—H12	119.6 (9)
C1—C2—C7	120.39 (11)	C11—C12—H12	121.7 (9)
C3—C2—C7	121.21 (12)	C12—C13—C14	121.92 (12)
C4—C3—C2	119.82 (12)	C12—C13—H13	120.1 (9)
C4—C3—C8	118.60 (13)	C14—C13—H13	118.0 (9)
C2—C3—C8	121.53 (13)	C13—C14—C9	119.23 (11)
C5—C4—C3	121.32 (12)	C13—C14—C15	119.71 (11)
C5—C4—H4	120.2 (10)	C9—C14—C15	121.00 (10)
C3—C4—H4	118.5 (10)	O1—C15—N2	121.19 (10)
C6—C5—C4	119.50 (13)	O1—C15—C14	123.33 (10)
C6—C5—H5	119.7 (10)	N2—C15—C14	115.47 (10)
C4—C5—H5	120.7 (10)	N3—C16—C17	120.80 (11)
C5—C6—C1	120.11 (13)	N3—C16—H16	122.0 (9)
C5—C6—H6	121.1 (11)	C17—C16—H16	117.0 (9)
C1—C6—H6	118.7 (11)	C18—C17—O2	109.77 (12)
C2—C7—H7A	110.2 (13)	C18—C17—C16	131.50 (13)
C2—C7—H7B	112.8 (13)	O2—C17—C16	118.59 (11)
H7A—C7—H7B	106.1 (17)	C17—C18—C19	106.60 (14)
C2—C7—H7C	112.1 (11)	C17—C18—H18	124.4 (11)
H7A—C7—H7C	108.4 (16)	C19—C18—H18	129.0 (11)
H7B—C7—H7C	106.9 (17)	C20—C19—C18	106.30 (13)
C3—C8—H8A	110.5 (11)	C20—C19—H19	126.7 (11)
C3—C8—H8B	111.0 (15)	C18—C19—H19	127.0 (11)
H8A—C8—H8B	108.1 (18)	C19—C20—O2	111.25 (13)
C3—C8—H8C	113.8 (13)	C19—C20—H20	131.6 (12)
H8A—C8—H8C	104.2 (17)	O2—C20—H20	117.1 (12)
			
C15—N2—N3—C16	177.81 (11)	C11—C12—C13—C14	0.5 (2)
C9—N1—C1—C6	−44.52 (19)	C12—C13—C14—C9	−1.49 (19)
C9—N1—C1—C2	139.08 (13)	C12—C13—C14—C15	−178.79 (12)
C6—C1—C2—C3	0.10 (18)	N1—C9—C14—C13	178.37 (11)
N1—C1—C2—C3	176.49 (11)	C10—C9—C14—C13	2.26 (17)
C6—C1—C2—C7	−177.09 (12)	N1—C9—C14—C15	−4.36 (18)
N1—C1—C2—C7	−0.70 (18)	C10—C9—C14—C15	179.53 (11)
C1—C2—C3—C4	1.90 (18)	N3—N2—C15—O1	−12.62 (17)
C7—C2—C3—C4	179.07 (12)	N3—N2—C15—C14	166.53 (10)
C1—C2—C3—C8	−175.36 (12)	C13—C14—C15—O1	157.42 (11)
C7—C2—C3—C8	1.81 (19)	C9—C14—C15—O1	−19.84 (18)
C2—C3—C4—C5	−1.98 (19)	C13—C14—C15—N2	−21.70 (16)
C8—C3—C4—C5	175.36 (13)	C9—C14—C15—N2	161.04 (11)
C3—C4—C5—C6	0.0 (2)	N2—N3—C16—C17	173.06 (11)
C4—C5—C6—C1	2.0 (2)	C20—O2—C17—C18	0.28 (15)
C2—C1—C6—C5	−2.09 (19)	C20—O2—C17—C16	−175.78 (12)
N1—C1—C6—C5	−178.41 (12)	N3—C16—C17—C18	177.42 (14)
C1—N1—C9—C10	−14.2 (2)	N3—C16—C17—O2	−7.54 (18)
C1—N1—C9—C14	169.88 (12)	O2—C17—C18—C19	−0.22 (16)
N1—C9—C10—C11	−178.26 (12)	C16—C17—C18—C19	175.15 (13)
C14—C9—C10—C11	−2.20 (19)	C17—C18—C19—C20	0.08 (17)
C9—C10—C11—C12	1.3 (2)	C18—C19—C20—O2	0.10 (18)
C10—C11—C12—C13	−0.4 (2)	C17—O2—C20—C19	−0.23 (16)

### 3-(2,3-Dimethylanilino)-N'-[(E)-(furan-2-yl)methylidene]benzohydrazide (I) Hydrogen-bond geometry (Å, º)

*Cg*1 and *Cg*2 are the centroids of the C17–C20/O2 and C1–C6 rings, respectively.

**Table:**

*D*—H···*A*	*D*—H	H···*A*	*D*···*A*	*D*—H···*A*
N1—H1···O1	0.910 (19)	1.977 (18)	2.7045 (14)	135.8 (15)
N2—H2···O1^i^	0.883 (18)	2.014 (18)	2.8458 (13)	156.4 (15)
C4—H4···*Cg*1^ii^	0.955 (17)	2.941 (17)	3.7248 (15)	140.1 (17)
C6—H6···O1^iii^	0.976 (18)	2.556 (18)	3.3434 (16)	137.7 (14)
C11—H11···*Cg*2^iv^	0.996 (16)	2.765 (16)	3.6231 (14)	144.8 (12)

Symmetry codes: (i) *x*, −*y*+1/2, *z*+1/2; (ii) −*x*+1, *y*+1/2, −*z*+1/2; (iii) −*x*+1, −*y*+1, −*z*+1; (iv) *x*, *y*, *z*+1.

### N'-[(E)-Benzylidene]-2-(2,3-dimethylanilino)-benzohydrazide (II) Crystal data

**Table:**

C_22_H_21_N_3_O	*F*(000) = 728
*M**_r_* = 343.42	*D*_x_ = 1.256 Mg m^−^^3^
Monoclinic, *P*2_1_/*c*	Cu *K*α radiation, λ = 1.54178 Å
*a* = 14.3493 (8) Å	Cell parameters from 9904 reflections
*b* = 15.7501 (9) Å	θ = 4.3–74.6°
*c* = 8.3737 (5) Å	µ = 0.62 mm^−^^1^
β = 106.285 (2)°	*T* = 150 K
*V* = 1816.55 (18) Å^3^	Block, pale yellow
*Z* = 4	0.19 × 0.13 × 0.08 mm

### N'-[(E)-Benzylidene]-2-(2,3-dimethylanilino)-benzohydrazide (II) Data collection

**Table:**

Bruker D8 VENTURE PHOTON 100 CMOS diffractometer	3665 independent reflections
Radiation source: INCOATEC IµS micro–focus source	3140 reflections with *I* > 2σ(*I*)
Mirror monochromator	*R*_int_ = 0.031
Detector resolution: 10.4167 pixels mm^-1^	θ_max_ = 74.6°, θ_min_ = 4.3°
ω scans	*h* = −17→16
Absorption correction: multi-scan (*SADABS*; Krause *et al.*, 2015)	*k* = −19→18
*T*_min_ = 0.86, *T*_max_ = 0.95	*l* = −10→9
13875 measured reflections	

### N'-[(E)-Benzylidene]-2-(2,3-dimethylanilino)-benzohydrazide (II) Refinement

**Table:**

Refinement on *F*^2^	Secondary atom site location: difference Fourier map
Least-squares matrix: full	Hydrogen site location: difference Fourier map
*R*[*F*^2^ > 2σ(*F*^2^)] = 0.037	All H-atom parameters refined
*wR*(*F*^2^) = 0.092	*w* = 1/[σ^2^(*F*_o_^2^) + (0.0409*P*)^2^ + 0.444*P*] where *P* = (*F*_o_^2^ + 2*F*_c_^2^)/3
*S* = 1.04	(Δ/σ)_max_ < 0.001
3665 reflections	Δρ_max_ = 0.17 e Å^−^^3^
320 parameters	Δρ_min_ = −0.15 e Å^−^^3^
0 restraints	Extinction correction: *SHELXL* 2016/6 (Sheldrick, 2015*b*), Fc^*^=kFc[1+0.001xFc^2^λ^3^/sin(2θ)]^-1/4^
Primary atom site location: structure-invariant direct methods	Extinction coefficient: 0.0033 (3)

### N'-[(E)-Benzylidene]-2-(2,3-dimethylanilino)-benzohydrazide (II) Special details

**Table:**

Geometry. All esds (except the esd in the dihedral angle between two l.s. planes) are estimated using the full covariance matrix. The cell esds are taken into account individually in the estimation of esds in distances, angles and torsion angles; correlations between esds in cell parameters are only used when they are defined by crystal symmetry. An approximate (isotropic) treatment of cell esds is used for estimating esds involving l.s. planes.
Refinement. Refinement of F^2^ against ALL reflections. The weighted R-factor wR and goodness of fit S are based on F^2^, conventional R-factors R are based on F, with F set to zero for negative F^2^. The threshold expression of F^2^ > 2sigma(F^2^) is used only for calculating R-factors(gt) etc. and is not relevant to the choice of reflections for refinement. R-factors based on F^2^ are statistically about twice as large as those based on F, and R- factors based on ALL data will be even larger.

### N'-[(E)-Benzylidene]-2-(2,3-dimethylanilino)-benzohydrazide (II) Fractional atomic coordinates and isotropic or equivalent isotropic displacement parameters (Å^2^)

**Table:**

	*x*	*y*	*z*	*U*_iso_*/*U*_eq_	
O1	0.44514 (6)	0.33126 (6)	0.43024 (9)	0.0300 (2)	
N1	0.62636 (8)	0.39413 (8)	0.53993 (14)	0.0392 (3)	
H1	0.5771 (13)	0.3712 (11)	0.458 (2)	0.051 (5)*	
N2	0.39227 (7)	0.26174 (7)	0.62519 (12)	0.0287 (2)	
H2	0.4042 (11)	0.2421 (10)	0.732 (2)	0.040 (4)*	
N3	0.30815 (7)	0.23802 (7)	0.50842 (12)	0.0278 (2)	
C1	0.70441 (8)	0.43293 (8)	0.49433 (14)	0.0301 (3)	
C2	0.75817 (9)	0.38475 (8)	0.41121 (14)	0.0301 (3)	
C3	0.83421 (9)	0.42441 (9)	0.36395 (15)	0.0330 (3)	
C4	0.85496 (10)	0.50931 (9)	0.40197 (17)	0.0386 (3)	
H4	0.9098 (13)	0.5351 (11)	0.367 (2)	0.052 (5)*	
C5	0.80049 (11)	0.55653 (9)	0.48198 (18)	0.0411 (3)	
H5	0.8132 (13)	0.6202 (12)	0.504 (2)	0.053 (5)*	
C6	0.72458 (10)	0.51889 (9)	0.52686 (16)	0.0365 (3)	
H6	0.6833 (13)	0.5527 (12)	0.578 (2)	0.054 (5)*	
C7	0.73544 (13)	0.29257 (9)	0.3725 (2)	0.0451 (4)	
H7A	0.7008 (18)	0.2847 (16)	0.256 (3)	0.095 (7)*	
H7B	0.7995 (19)	0.2567 (16)	0.388 (3)	0.096 (8)*	
H7C	0.6979 (15)	0.2671 (13)	0.443 (3)	0.072 (6)*	
C8	0.89350 (12)	0.37677 (12)	0.2713 (2)	0.0490 (4)	
H8A	0.8504 (15)	0.3577 (13)	0.155 (3)	0.068 (6)*	
H8B	0.9499 (16)	0.4120 (13)	0.259 (2)	0.074 (6)*	
H8C	0.9197 (17)	0.3238 (15)	0.328 (3)	0.083 (7)*	
C9	0.60826 (8)	0.39908 (8)	0.69193 (14)	0.0300 (3)	
C10	0.67446 (9)	0.43734 (9)	0.83041 (16)	0.0356 (3)	
H10	0.7358 (12)	0.4613 (10)	0.8156 (19)	0.045 (4)*	
C11	0.65461 (10)	0.44332 (9)	0.98135 (16)	0.0391 (3)	
H11	0.7018 (12)	0.4732 (10)	1.073 (2)	0.045 (4)*	
C12	0.56938 (10)	0.41042 (9)	1.00387 (16)	0.0386 (3)	
H12	0.5526 (12)	0.4186 (11)	1.110 (2)	0.053 (5)*	
C13	0.50518 (9)	0.36998 (8)	0.87248 (15)	0.0323 (3)	
H13	0.4448 (11)	0.3485 (10)	0.8855 (18)	0.037 (4)*	
C14	0.52311 (8)	0.36209 (8)	0.71679 (14)	0.0273 (3)	
C15	0.45190 (8)	0.31809 (8)	0.57935 (14)	0.0259 (2)	
C16	0.25463 (8)	0.18507 (8)	0.55850 (14)	0.0283 (3)	
H16	0.2745 (11)	0.1595 (10)	0.6724 (19)	0.039 (4)*	
C17	0.15643 (8)	0.16629 (8)	0.45380 (14)	0.0270 (2)	
C18	0.11897 (9)	0.20627 (8)	0.30028 (15)	0.0325 (3)	
H18	0.1624 (12)	0.2442 (11)	0.258 (2)	0.046 (4)*	
C19	0.02375 (10)	0.19387 (9)	0.20915 (17)	0.0387 (3)	
H19	−0.0003 (12)	0.2241 (11)	0.098 (2)	0.051 (5)*	
C20	−0.03663 (9)	0.14166 (9)	0.26957 (17)	0.0377 (3)	
H20	−0.1059 (13)	0.1325 (11)	0.205 (2)	0.050 (4)*	
C21	−0.00080 (9)	0.10191 (9)	0.42071 (17)	0.0373 (3)	
H21	−0.0449 (13)	0.0660 (11)	0.467 (2)	0.051 (4)*	
C22	0.09561 (9)	0.11354 (9)	0.51326 (16)	0.0333 (3)	
H22	0.1219 (11)	0.0868 (10)	0.6234 (19)	0.038 (4)*	

### N'-[(E)-Benzylidene]-2-(2,3-dimethylanilino)-benzohydrazide (II) Atomic displacement parameters (Å^2^)

**Table:**

	*U*^11^	*U*^22^	*U*^33^	*U*^12^	*U*^13^	*U*^23^
O1	0.0270 (4)	0.0408 (5)	0.0213 (4)	−0.0033 (3)	0.0051 (3)	−0.0017 (3)
N1	0.0290 (5)	0.0598 (8)	0.0297 (5)	−0.0158 (5)	0.0096 (4)	−0.0104 (5)
N2	0.0229 (5)	0.0392 (6)	0.0212 (5)	−0.0030 (4)	0.0019 (4)	0.0015 (4)
N3	0.0219 (5)	0.0362 (6)	0.0235 (5)	−0.0016 (4)	0.0034 (4)	−0.0018 (4)
C1	0.0235 (5)	0.0390 (7)	0.0261 (6)	−0.0033 (5)	0.0041 (4)	0.0003 (5)
C2	0.0309 (6)	0.0328 (6)	0.0253 (6)	−0.0003 (5)	0.0053 (5)	0.0017 (5)
C3	0.0282 (6)	0.0424 (7)	0.0281 (6)	0.0030 (5)	0.0076 (5)	0.0056 (5)
C4	0.0333 (7)	0.0454 (8)	0.0364 (7)	−0.0080 (6)	0.0085 (5)	0.0067 (6)
C5	0.0463 (8)	0.0333 (7)	0.0417 (7)	−0.0066 (6)	0.0092 (6)	0.0012 (6)
C6	0.0354 (7)	0.0362 (7)	0.0368 (7)	0.0037 (5)	0.0083 (5)	−0.0027 (5)
C7	0.0632 (10)	0.0346 (7)	0.0402 (8)	−0.0068 (7)	0.0189 (7)	−0.0025 (6)
C8	0.0463 (8)	0.0616 (10)	0.0453 (9)	0.0114 (8)	0.0229 (7)	0.0053 (7)
C9	0.0248 (5)	0.0366 (7)	0.0263 (6)	−0.0003 (5)	0.0034 (4)	−0.0009 (5)
C10	0.0292 (6)	0.0416 (7)	0.0306 (6)	−0.0046 (5)	−0.0005 (5)	−0.0013 (5)
C11	0.0416 (7)	0.0412 (7)	0.0268 (6)	−0.0047 (6)	−0.0030 (5)	−0.0025 (5)
C12	0.0467 (7)	0.0436 (8)	0.0234 (6)	−0.0018 (6)	0.0065 (5)	−0.0032 (5)
C13	0.0336 (6)	0.0367 (7)	0.0260 (6)	−0.0004 (5)	0.0074 (5)	−0.0008 (5)
C14	0.0246 (5)	0.0329 (6)	0.0226 (5)	0.0012 (5)	0.0037 (4)	−0.0007 (4)
C15	0.0205 (5)	0.0326 (6)	0.0237 (5)	0.0029 (4)	0.0050 (4)	0.0003 (4)
C16	0.0260 (6)	0.0352 (6)	0.0236 (5)	0.0009 (5)	0.0066 (4)	0.0004 (5)
C17	0.0251 (6)	0.0307 (6)	0.0252 (5)	0.0008 (5)	0.0070 (4)	−0.0030 (4)
C18	0.0300 (6)	0.0369 (7)	0.0292 (6)	0.0000 (5)	0.0057 (5)	0.0012 (5)
C19	0.0330 (7)	0.0451 (8)	0.0326 (7)	0.0035 (6)	0.0001 (5)	0.0003 (6)
C20	0.0244 (6)	0.0447 (8)	0.0402 (7)	0.0007 (5)	0.0028 (5)	−0.0106 (6)
C21	0.0298 (6)	0.0425 (7)	0.0411 (7)	−0.0077 (6)	0.0123 (5)	−0.0075 (6)
C22	0.0306 (6)	0.0381 (7)	0.0306 (6)	−0.0049 (5)	0.0076 (5)	−0.0009 (5)

### N'-[(E)-Benzylidene]-2-(2,3-dimethylanilino)-benzohydrazide (II) Geometric parameters (Å, º)

**Table:**

O1—C15	1.2424 (14)	C9—C10	1.4115 (17)
N1—C9	1.3707 (16)	C9—C14	1.4208 (17)
N1—C1	1.4189 (16)	C10—C11	1.3745 (19)
N1—H1	0.908 (18)	C10—H10	0.997 (16)
N2—C15	1.3607 (16)	C11—C12	1.389 (2)
N2—N3	1.3750 (13)	C11—H11	0.990 (16)
N2—H2	0.913 (16)	C12—C13	1.3770 (18)
N3—C16	1.2814 (16)	C12—H12	0.988 (17)
C1—C6	1.3954 (19)	C13—C14	1.4033 (16)
C1—C2	1.3987 (17)	C13—H13	0.965 (16)
C2—C3	1.4068 (17)	C14—C15	1.4796 (15)
C2—C7	1.5034 (19)	C16—C17	1.4659 (16)
C3—C4	1.387 (2)	C16—H16	1.000 (15)
C3—C8	1.5030 (19)	C17—C22	1.3941 (17)
C4—C5	1.381 (2)	C17—C18	1.3966 (17)
C4—H4	1.001 (17)	C18—C19	1.3793 (18)
C5—C6	1.382 (2)	C18—H18	0.997 (17)
C5—H5	1.026 (18)	C19—C20	1.389 (2)
C6—H6	0.980 (18)	C19—H19	1.013 (18)
C7—H7A	0.97 (3)	C20—C21	1.376 (2)
C7—H7B	1.06 (3)	C20—H20	1.000 (18)
C7—H7C	0.99 (2)	C21—C22	1.3952 (18)
C8—H8A	1.04 (2)	C21—H21	1.004 (18)
C8—H8B	1.01 (2)	C22—H22	0.988 (15)
C8—H8C	0.98 (2)		
			
C9—N1—C1	126.50 (11)	C11—C10—C9	121.30 (12)
C9—N1—H1	114.2 (11)	C11—C10—H10	120.4 (9)
C1—N1—H1	118.4 (11)	C9—C10—H10	118.3 (9)
C15—N2—N3	118.21 (10)	C10—C11—C12	121.11 (12)
C15—N2—H2	122.4 (10)	C10—C11—H11	118.3 (9)
N3—N2—H2	119.3 (10)	C12—C11—H11	120.6 (9)
C16—N3—N2	115.55 (10)	C13—C12—C11	118.77 (12)
C6—C1—C2	120.68 (11)	C13—C12—H12	120.0 (10)
C6—C1—N1	120.10 (12)	C11—C12—H12	121.2 (10)
C2—C1—N1	119.17 (12)	C12—C13—C14	121.83 (12)
C1—C2—C3	118.60 (12)	C12—C13—H13	119.5 (9)
C1—C2—C7	120.96 (12)	C14—C13—H13	118.6 (9)
C3—C2—C7	120.44 (12)	C13—C14—C9	119.28 (11)
C4—C3—C2	119.79 (12)	C13—C14—C15	119.84 (11)
C4—C3—C8	118.91 (13)	C9—C14—C15	120.85 (10)
C2—C3—C8	121.30 (13)	O1—C15—N2	120.95 (10)
C5—C4—C3	121.08 (12)	O1—C15—C14	123.08 (10)
C5—C4—H4	121.5 (10)	N2—C15—C14	115.96 (10)
C3—C4—H4	117.4 (10)	N3—C16—C17	119.96 (11)
C4—C5—C6	119.84 (13)	N3—C16—H16	122.5 (9)
C4—C5—H5	121.3 (9)	C17—C16—H16	117.3 (9)
C6—C5—H5	118.8 (10)	C22—C17—C18	118.65 (11)
C5—C6—C1	119.96 (12)	C22—C17—C16	119.98 (11)
C5—C6—H6	120.4 (10)	C18—C17—C16	121.12 (11)
C1—C6—H6	119.6 (10)	C19—C18—C17	120.75 (12)
C2—C7—H7A	111.1 (15)	C19—C18—H18	120.5 (9)
C2—C7—H7B	111.2 (13)	C17—C18—H18	118.7 (9)
H7A—C7—H7B	103.8 (19)	C18—C19—C20	120.31 (12)
C2—C7—H7C	112.7 (12)	C18—C19—H19	118.0 (10)
H7A—C7—H7C	109.5 (19)	C20—C19—H19	121.7 (10)
H7B—C7—H7C	108.2 (18)	C21—C20—C19	119.62 (12)
C3—C8—H8A	110.6 (11)	C21—C20—H20	119.3 (10)
C3—C8—H8B	111.4 (12)	C19—C20—H20	121.0 (10)
H8A—C8—H8B	110.3 (15)	C20—C21—C22	120.51 (12)
C3—C8—H8C	111.8 (13)	C20—C21—H21	119.9 (10)
H8A—C8—H8C	104.6 (17)	C22—C21—H21	119.6 (10)
H8B—C8—H8C	107.9 (18)	C17—C22—C21	120.16 (12)
N1—C9—C10	121.87 (11)	C17—C22—H22	118.3 (9)
N1—C9—C14	120.49 (10)	C21—C22—H22	121.5 (9)
C10—C9—C14	117.58 (11)		
			
C15—N2—N3—C16	−179.78 (11)	C12—C13—C14—C9	−2.03 (19)
C9—N1—C1—C6	−49.49 (19)	C12—C13—C14—C15	−179.98 (12)
C9—N1—C1—C2	132.87 (14)	N1—C9—C14—C13	−178.39 (12)
C6—C1—C2—C3	1.20 (17)	C10—C9—C14—C13	4.26 (18)
N1—C1—C2—C3	178.83 (11)	N1—C9—C14—C15	−0.46 (18)
C6—C1—C2—C7	−178.68 (12)	C10—C9—C14—C15	−177.81 (11)
N1—C1—C2—C7	−1.05 (18)	N3—N2—C15—O1	−18.17 (17)
C1—C2—C3—C4	0.75 (18)	N3—N2—C15—C14	161.30 (10)
C7—C2—C3—C4	−179.36 (12)	C13—C14—C15—O1	156.06 (12)
C1—C2—C3—C8	−178.75 (12)	C9—C14—C15—O1	−21.86 (18)
C7—C2—C3—C8	1.13 (19)	C13—C14—C15—N2	−23.40 (16)
C2—C3—C4—C5	−1.70 (19)	C9—C14—C15—N2	158.68 (11)
C8—C3—C4—C5	177.82 (13)	N2—N3—C16—C17	169.95 (10)
C3—C4—C5—C6	0.7 (2)	N3—C16—C17—C22	−175.28 (12)
C4—C5—C6—C1	1.3 (2)	N3—C16—C17—C18	−1.04 (18)
C2—C1—C6—C5	−2.25 (19)	C22—C17—C18—C19	−0.02 (19)
N1—C1—C6—C5	−179.86 (12)	C16—C17—C18—C19	−174.33 (12)
C1—N1—C9—C10	−7.4 (2)	C17—C18—C19—C20	0.4 (2)
C1—N1—C9—C14	175.32 (12)	C18—C19—C20—C21	−0.3 (2)
N1—C9—C10—C11	178.76 (13)	C19—C20—C21—C22	−0.1 (2)
C14—C9—C10—C11	−3.9 (2)	C18—C17—C22—C21	−0.45 (19)
C9—C10—C11—C12	1.2 (2)	C16—C17—C22—C21	173.93 (12)
C10—C11—C12—C13	1.2 (2)	C20—C21—C22—C17	0.5 (2)
C11—C12—C13—C14	−0.7 (2)		

### N'-[(E)-Benzylidene]-2-(2,3-dimethylanilino)-benzohydrazide (II) Hydrogen-bond geometry (Å, º)

*Cg*1 and *Cg*3 are the centroids of the C1–C6 and C17–C22 benzene rings, respectively.

**Table:**

*D*—H···*A*	*D*—H	H···*A*	*D*···*A*	*D*—H···*A*
N1—H1···O1	0.908 (18)	1.946 (18)	2.6920 (13)	138.2 (15)
N2—H2···O1^i^	0.913 (16)	1.974 (17)	2.8564 (13)	162.0 (14)
C4—H4···*Cg*3^ii^	1.001 (17)	2.796 (17)	3.6141 (15)	139.4 (13)
C6—H6···O1^iii^	0.980 (18)	2.583 (19)	3.4815 (16)	152.5 (13)
C20—H20···*Cg*1^iv^	1.000 (18)	2.838 (17)	3.6644 (15)	140.5 (13)

Symmetry codes: (i) *x*, −*y*+1/2, *z*+1/2; (ii) −*x*+1, *y*+1/2, −*z*+1/2; (iii) −*x*+1, −*y*+1, −*z*+1; (iv) *x*−1, −*y*−1/2, *z*−3/2.
